# *Neisseria gonorrhoeae* NGO2105 Is an Autotransporter Protein Involved in Adhesion to Human Cervical Epithelial Cells and *in vivo* Colonization

**DOI:** 10.3389/fmicb.2020.01395

**Published:** 2020-06-25

**Authors:** Jian Huang, Qing Zhang, Jie Chen, Tao Zhang, Zehui Chen, Zuyi Chen, Jianru Yang, Yongxiang Wang, Zongsu Min, Meirong Huang, Xun Min

**Affiliations:** ^1^Department of Laboratory Medicine, Affiliated Hospital of Zunyi Medical University, Zunyi, China; ^2^Zunyi Maternal and Child Health Hospital, Zunyi, China; ^3^Department of Blood Transfusion, Affiliated Hospital of Zunyi Medical University, Zunyi, China

**Keywords:** *Neisseria gonorrhoeae*, NGO2105, autotransporter, adhesion, colonization

## Abstract

Autotransporters are important virulence factors in the outer membrane of gram-negative bacteria. Although several autotransporters have been identified in *Neisseria meningitidis*, only IgA1 protease has been identified in *Neisseria gonorrhoeae*. A sequence analysis showed a marked difference in the distribution of autotransporters between the two strains. It has been speculated that only two autotransporters, the IgA1 protease and the NGO2105 protein, might be encoded by *N. gonorrhoeae*. Here, we describe the identification of NGO2105, a new autotransporter in *N. gonorrhoeae*. A sequence alignment showed that NGO2105 is highly similar to the adhesion and penetration protein (App) in *N. meningitidis*. We found that NGO2105 is exported to the outer membrane, cleaved and released into the culture supernatant by endogenous serine protease activity in *N. gonorrhoeae* and *E. coli*. The site-directed mutagenesis of S267A in the predicted enzyme catalytic triad abolished autoproteolytic cleavage to allow secretion. The NGO2105 β-barrel shows the ability to translocate the heterologous Hbp passenger domain. NGO2105 is involved in gonococcal adherence to and invasion into human cervical epithelial cells. Furthermore, antibodies raised against NGO2105 are able to block gonococcal adherence to human cervical epithelial cells. The Δ*ngo2105* mutant and anti-NGO2105 antiserum significantly attenuated the colonization of *N. gonorrhoeae* in mice. Collectively, our results suggest that the newly identified serine protease autotransporter NGO2105 represents a novel virulence factor of gonococcus and a potential vaccine target.

## Introduction

*Neisseria gonorrhoeae* is the causative pathogen of the sexually transmitted disease gonorrhea and causes mucosal infections of the genital tract, pharynx, rectum, and conjunctiva (Peterman et al., 2016; Semchenko and Seib, 2016). In women, the most common manifestation is cervicitis, but approximately 50–80% of patients experience asymptomatic infections (Farley et al., 2003). If not treated, more than 45% of patients can develop pelvic inflammation, hysteritis, salpingitis, or ovarian inflammation (Edwards and Apicella, 2004). In men, the typical symptoms of gonorrhea include urethral mucopurulent discharge and dysuria (Handsfield et al., 1974; Farley et al., 2003). In addition, *N. gonorrhoeae* infection also increases the risk of acquiring and transmitting HIV (Feily and Namazi, 2010; Jarvis and Chang, 2012). Antibiotics have always been an effective treatment for gonorrhea, but similar to the results obtained with most bacteria, *N. gonorrhoeae* strains that exhibit resistance to the prescribed drugs have emerged (Unemo and Shafer, 2014). Due the increased resistance of *N. gonorrhoeae* to various antibiotics, particularly the emergence and spread of strains that are highly resistant to broad-spectrum cephalosporins, no drugs might be available for *N. gonorrhoeae* treatment (Bolan et al., 2012; Blomquist et al., 2014; Tuddenham and Ghanem, 2015). Therefore, *N. gonorrhoeae* was listed as an “urgent threat event” by the World Health Organization (WHO) (Blomquist et al., 2014). For these reasons, the search for effective treatment strategies, such as new drugs and vaccines, has become more urgent (Russell et al., 2019). Further exploration of the pathogenic molecules of gonorrhoeae has become even more important for the development of new therapeutic targets.

Gram-negative bacteria have evolved different secretion systems for protein secretion, and these have been classified as types I–IX secretion systems. The proteins that form part of the type V secretion system are usually called autotransporters (Meuskens et al., 2019), and these proteins constitute a large class of proteins that are found in the outer membrane of gram-negative bacteria and have a variety of virulence functions, such as adherence, invasion, protease activity, and cytotoxicity (Pokharel et al., 2019). According to their different structural characteristics and domain organization, type V secretory systems are further divided into different subtypes, ranging from type Va to type Vf (Meuskens et al., 2019). The autotransporters of type Va secretory systems, which are commonly known as classical autotransporters, consist of an N-terminal signal sequence, a secreted passenger domain, and a C-terminal β-barrel (translocator) domain (Henderson et al., 1998). During the process of secretion, the N-terminal signal sequence directs the protein to the Sec machinery for transport across the inner membrane. Subsequently, the β-barrel is inserted into the outer membrane, where it is thought to form a pore through which the functional passenger domain passes (Pavlova et al., 2013; Leyton et al., 2014). The passenger domain is then localized on the bacterial surface or released into the extracellular environment via proteolytic cleavage (Spahich and St Geme, 2011; Meuskens et al., 2019). This mechanism of secretion was first described for the IgA1 protease of *N. gonorrhoeae*, and more autotransporters have been found in other bacteria (Pohlner et al., 1987). Eight autotransporters have been identified in *Neisseria meningitidis*, whereas in *N. gonorrhoeae*, only the IgA1 protease has been identified (van Ulsen and Tommassen, 2006; Tommassen and Arenas, 2017). A genome sequence comparison revealed significant differences in the distribution of autotransporters between *N. meningitidis* and *N. gonorrhoeae*. On the one hand, the *N. gonorrhoeae* genome contains some pseudogenes that are homologous to *N. meningitidis* autotransporter genes, such as NGO1155/6 (Ata-1), NGO0985 (AutB), and NGO0694 (Ata-3), but their ORFs are disrupted by termination codons or deletions, which appear to be dispensable for *N. gonorrhoeae* (van Ulsen and Tommassen, 2006). On the other hand, some *N. meningitidis* autotransporter homologs have not been found in the *N. gonorrhoeae* genome, and these include NhhA, IhhA, IhhB, NalP, and NadA (van Ulsen and Tommassen, 2006). In addition, the *N. gonorrhoeae* genome encodes only two apparently functional type Va autotransporters: the IgA1 protease and the NGO2105 protein. However, the biological function of NGO2105 in *N. gonorrhoeae* has not been identified. A sequence alignment showed that *N. gonorrhoeae* NGO2105 is highly similar to the adhesion and penetration protein (App) of *N. meningitidis*. App is a serine protease autotransporter whose passenger domain can release the extracellular environment through autoproteolysis (Hadi et al., 2001). App can mediate the adhesion of *N. meningitidis* to the human epithelial cell line Chang (Serruto et al., 2003). The expression of App protein appears to confer significant virulence during pathogenesis *in vivo*, as demonstrated by the finding that mice infected with the App-deficient meningococcal mutants survived better than the wild-type mice (Khairalla et al., 2015). App can proteolytically cleave core histone H3 and induce the apoptosis of dendritic cells through a caspase-dependent mechanism (Khairalla et al., 2015).

The aim of this study was to determine whether NGO2105 is a serine proteinase autotransporter expressed in *N. gonorrhoeae* by analyzing the surface localization, secretion, and autoproteolytic cleavage of NGO2105. In addition, we further evaluated the role of NGO2105 in gonococcal pathogenesis through *in vivo* and *in vitro* experiments and evaluated the protective effects of its antibody.

## Materials and Methods

### Bacterial Strains and Growth Conditions

All *N. gonorrhoeae* strains used in this study were in the background of *N. gonorrhoeae* strain FA1090. The *N. gonorrhoeae* strains were grown on gonococcal base liquid (GCBL) medium or GCB plates at 37°C in the presence of 5% CO_2_. The *Escherichia coli* strains DH5α, BL21(DE3) and C41(DE3) were used in this study and grown in lysogeny broth (LB) with shaking or on LB agar at 37°C. When appropriate, the GCB and GCBL used for *N. gonorrhoeae* growth were supplemented with the antibiotic spectinomycin (100 μg/mL). For *E. coli*, antibiotics were used at the following concentrations: kanamycin (50 μg/mL) or ampicillin (100 μg/mL). When required, gene expression was induced with isopropyl-β-D-thiogalactoside (IPTG).

### Bioinformatics Analysis

The NCBI CDD Search server was used to identify the conserved domain. The NCBI BLAST server and Clustal W 2.1 software were used for sequence alignment. The three-dimensional structure of NGO2105 was homology-modeled using Swiss-model^1^ (Waterhouse et al., 2018).

### DNA Manipulations and Genetic Techniques

Chromosomal DNA of *N. gonorrhoeae* strain FA1090 was used as a template for PCR. All the primers used in the PCR assay are shown in Table 1. The full-length *ngo2105* sequence and a truncated *ngo2105p* sequence (encoding the passenger domain of NGO2105) were obtained by PCR. The PCR products were digested with *EcoR*I/*Hind*III and inserted into the pET28a (Novagen) and pCold-TF (Takara) vectors to obtain the pET28a-NGO2105, pCold-TF-NGO2105, and pCold-TF-NGO2105P constructs.

**TABLE 1:** Primers used in this study.

**Primer**	**Sequence (5′–3′)**
NGO2105-F	CCGGAATTCATGAAA ACAACCGACAAACGGACAA
NGO2105-R	CCCAAGCTTTTACCAGC GGTAGCCTAATTTGATG
NGO2105P-F	CCGGAATTCGGACACACTTATTTCGGCATCAACT
NGO2105P-R	CCCAAGCTTATGTGGT TCGTAGAATACTGAATGG
△*ngo2105-*F1	ATGCCGTCTGAAG GTTCAGCAGCATCTCCATCA CTAC
△*ngo2105-*R1	TTGAA CCAGCTATTTTTCCTTATCTGACGGGATTC
△*ngo2105-*F2	AGGAAAAATAGCTGGTTCAAGCCAAAGGGGAAAAC
△*ngo2105-*R2	TGACCACCCGCTTCCTAAATGATTG
△*ngo2105*::*2105-*F	CCGCTTAAGATGAAAACAACCGACAAACGGACAA
△*ngo2105*::*2105-*R	CCCCCCGGGTTACCAGCGGTAGCCTAATTTGATG
NGO2105S267A-F	CTCATTTGGCGACgctGGCTCACCAATGTTTATCTATGATG
NGO2105S267A-R	CATCATAGATAAACATTGGTGAGCCagcGTCGCCAAATGAG
Hbp passenger-F	GCCGGAATTCATGAACAGA ATTTATTCTCTTC
Hbp passenger-R	GTCAGCGTGTTGAAACGGGAGCTGATGTGCATGAATG
NGO2105β-F	CATTCATGCACATCAGCTCCCGTTTCAACACGCTGAC
NGO2105β-R	CCCAAGCTTTTACCAGCGGTAGC CTAATT

Because the DNA uptake sequence (DUS) increased the natural transformation efficiency of *N. gonorrhoeae* (Duffin and Seifert, 2010), for constructing the deletion mutant of *ngo2105* (Δ*ngo2105*) in *N. gonorrhoeae* strain FA1090, a DUS sequence (GCCGTCTGAA) was designed at the 5′-terminus of the primer Δ*ngo2105*F1 to facilitate transformation. The 5′ flanking fragment of *ngo2105* was amplified by PCR using the primers Δ*ngo2105*F1/R1, and a 577bp fragment of the 3′-terminal sequence of *ngo2105* was amplified by PCR using Δ*ngo2105*F2/R2. The two PCR products were linked by overlap extension PCR, and the ligation product was transformed into *N. gonorrhoeae* FA1090 by spot transformation on plates as previously described (Dillard, 2011). The coding sequence of *ngo2105* could be deleted after homologous recombination. The positive transformants were screened by PCR. The complementation construct (Δ*ngo2105*::*2105*) was generated by cloning *ngo2105* into the pCTS32 plasmid between the *Afl*II and *Sma*I restriction sites as previously described (Steichen et al., 2008). The construct was linearized with *Nco*I and transformed into the FA1090Δ*ngo2105* strains using standard protocols. The FA1090Δ*ngo2105* and FA1090Δ*ngo2105*::*2105* constructs were confirmed by DNA sequencing, qRT-PCR and Western blot analysis.

To construct the S267A point mutation of NGO2105, we used site-directed mutagenesis method as previously described (Fisher and Pei, 1997), in which the templates were the pET28a-NGO2105 and pCTS32-NGO2105 plasmids. The PCR products were digested by *Dpn*I and transformed into *E. coli* DH5α. The positive transformants were confirmed by DNA sequencing. The pET28a-NGO2105S267A and pCTS32-NGO2105S267A vectors were transformed into *E. coli* C41(DE3) and FA1090Δ*ngo2105*, respectively, as described above.

**FIGURE 1:**
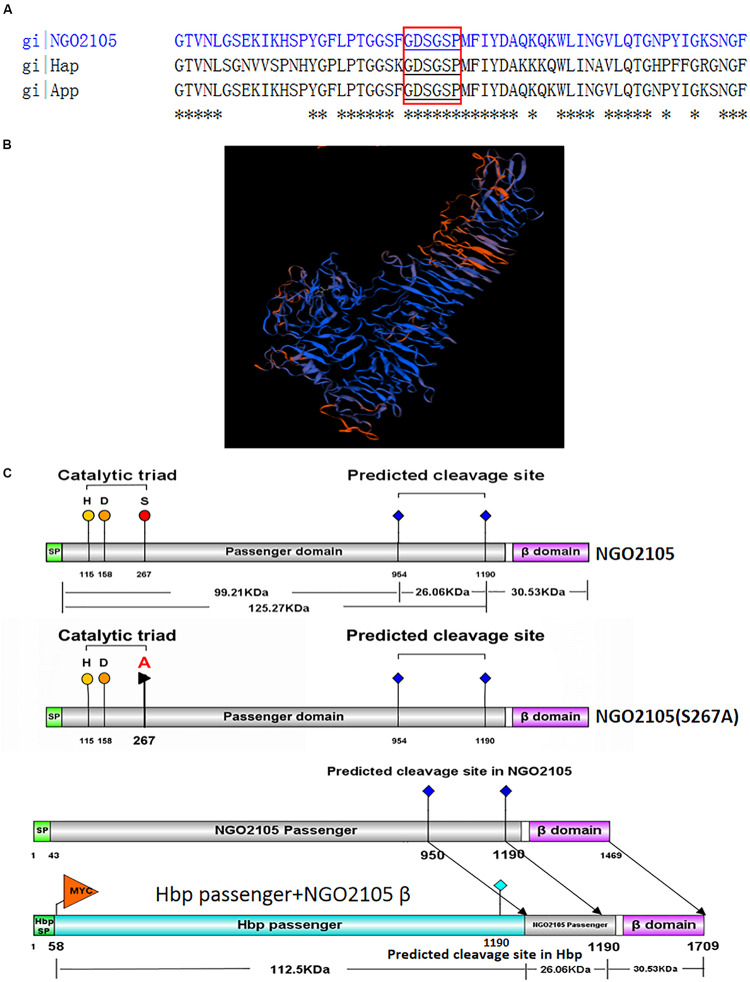
*In silico* analysis of NGO2105. **(A)** Sequence alignment of NGO2105 (strain FA1090) with *H. influenzae* Hap and the *N. meningitidis* App. Asterisks (*) denote positions in the sequence with fully conserved residues. The conserved serine protease motif (GDSGSP) is highlighted in red boxes. **(B)** Homology model of NGO2105. The panel shows the predicted three-dimensional structure of NGO2105 obtained with the homology model. **(C)** Schematic representation of NGO2105 features and the constructs used in this study. The upper panel shows the NGO2105 features, which consist of an N-terminal signal peptide (SP, light green), the putative passenger domain (gray) and the predicted C-terminal β-barrel (purple). H_115_, D_158_, and S_267_ represent the predicted serine protease catalytic triad. _953_NTL_955_ and _1189_NSG_1191_ represent the predicted cleavage sites. The middle panel shows the mutation of serine 267 to alanine. The lower panel shows the construct of Hbp passenger-NGO2105β, which consists of the signal peptide and the passenger domain from *E. coli* Hbp and the β-barrel and partial passenger domain (containing the cleavage site) from *N. gonorrhoeae* NGO2105.

To construct the recombinant plasmid pET28a-Hbp passenger-NGO2105β, a modified *hbp passenger* gene sequence was synthesized by Fenghui Biotechnology Co., Ltd. (Hunan, China) as described in the lower panel of Figure 1C; this gene sequence consisted of the N-terminal signal peptide of Hbp, a gene sequence encoding the Myc tag and a gene sequence encoding the passenger domain of the Hbp protein. The gene sequence encoding the β-barrel of NGO2105 was then amplified by PCR using the primers *ngo2015β*F/R. Both the *hbp-passenger* and *ngo2105β* gene fragments were linked by overlap extension PCR, and the ligation product was cloned into the pET28a plasmid to construct the recombinant vector pET28a-Hbp passenger-NGO2105β. As a control, the Hbp-passenger sequence was inserted into the pET28a vector to generate the pET28a-Hbp-passenger. Both recombinant plasmids were verified by sequencing and then transformed into *E. coli* C41(DE3).

### Recombinant Protein Expression, Purification, and Preparation of Polyclonal Antisera

For the expression of full-length NGO2105 and NGO2105P, the recombinant pCold -TF-NGO2105 and pCold -TF-NGO2105P plasmids were transformed into *E. coli* BL21(DE3) for protein induction. After the expression strains were cultured to an OD_600_ of 0.6, 0.2 mM IPTG was added, and the cultures were further incubated overnight at 15°C. The bacterial cells were pelleted, resuspended in 1 × binding buffer (300 mmol/L NaCl and 10 mmol/L PBS, pH 8.0) and lysed by sonication. The full-length NGO2105 protein was expressed in the form of inclusion bodies. Inclusion bodies were collected by centrifugation at 10,000 g for 20 min. Wash the inclusion body 3 times with inclusion body washing solution (20 mM Tris, 1 mM EDTA, 2M urea, 1M NaCl, 1% Triton X-100, pH8.0). The inclusion bodies were dissolved in dissolution buffer (20 mM Tris, 5 mM DTT, 8 M urea, pH 8.0) and left overnight at 4°C. The above solution was added dropwise to the buffer solution (20 mM Tris-HCL, 5 mM EDTA, pH7.8) and gradually diluted. Then, the protein solution was put into a dialysis bag and dialyzed against PBS pH7.4 solution overnight. The NGO2105P protein was expressed in soluble form. The two proteins were purified using a Ni^2+^-nitrilotriacetic acid (Ni-NTA) affinity chromatography column (GE Healthcare, Little Chalfont, United Kingdom). The protein supernatant-resin mixture was washed with 10 column volumes of phosphate buffer containing 20 mM imidazole. After washing, the recombinant protein was eluted in phosphate buffer containing 300 mM imidazole, concentrated and dialyzed against sterile PBS to remove imidazole. The protein purity was detected by SDS-PAGE electrophoresis. BALB/c mice were immunized with the two purified proteins to prepare anti-NGO2105 and anti-NGO2105P antiserum. Groups of five 6-week-old BALB/c female mice (Laboratory Animal Center, Changsha, China) were immunized subcutaneously with 20 μg of recombinant protein at day 1 (with an equal volume Freund’s complete adjuvant, Sigma-Aldrich) and at days 14 and 28 (with an equal volume of Freund’s incomplete adjuvant, Sigma-Aldrich). Blood samples were collected on day 42, and the serum titers were determined by ELISA.

### Preparation of Whole-Cell Protein and Supernatant Protein

For preparation of the proteins of *N. gonorrhoeae* FA1090, FA1090Δ*ngo2105*, FA1090Δ*ngo2105::2105*, and FA1090Δ*ngo2105::2105*S267A, these strains were grown overnight at 37°C on GCB plates with 5% CO_2_. The clones were collected in GCBL medium and the initial bacterial density was adjusted to an OD_540_ of 0.2. The cultures were then grown to an OD_540_ of 0.8 at 37°C. The bacterial pellet and the culture supernatant were separated by centrifugation at 13,000 g for 10 min. To prepare the whole-cell lysates of the above-mentioned strains, the same amount of bacterial pellet was resuspended in PBS and boiled for 20 min. For precipitation of the proteins in the culture supernatant, 5 mL of the above-described culture supernatants was collected and precipitated using the methanol-chloroform method as described previously (Wessel and Flugge, 1984).

For preparation of the proteins of *E. coli* C41(DE3)/pET28a-NGO2105 and *E. coli* C41(DE3)/pET28a-NGO2105S267A, positive clones were selected and inoculated in LB medium with antibiotics at 37°C until an OD_600_ of 0.5. IPTG (1 mM) was added, and the cultures were then incubated at 37°C for 4 h. The bacterial density was adjusted to the same level. To prepare the whole-cell lysates of the above-mentioned strains, 1 mL of the bacterial culture was centrifuged at 13,000 g for 10 min, resuspended in the same amount of PBS and boiled for 20 min. To precipitate the proteins in the culture supernatants, 5 mL of the above-described cultures with the same bacterial density was centrifuged at 13,000 g for 10 min, and the supernatant was collected and precipitated using the methanol-chloroform method as described previously (Wessel and Flugge, 1984). The obtained pellet was resuspended in 1× sample loading buffer. All whole-cell lysates and supernatant proteins were used for subsequent Western blot analysis.

### Western Blot Analysis of NGO2105 Expression in *N. gonorrhoeae* and *E. coli*

The whole-cell lysates and precipitated supernatant proteins obtained from different *N. gonorrhoeae* and *E. coli* C41(DE3) strains were separated by SDS–PAGE using 10% polyacrylamide gels, and immunoblotting was performed with a 1:1000 dilution of anti-NGO2105 or anti-NGO2105P polyclonal antiserum as the primary antibody. Horseradish peroxidase (HRP)-conjugated goat anti-mouse IgG was used as the secondary antibody. The bands were detected using chemiluminescent substrate. Duplicate gels were run and stained with Coomassie brilliant blue to confirm equal loading of the samples.

### Flow Cytometry Analysis of the NGO2105 Surface Exposure in *N. gonorrhoeae* and *E. coli*

Cultures of *N. gonorrhoeae* FA1090, FA1090Δ*ngo2105*, FA1090Δ*ngo2105::2105*, and FA1090Δ*ngo2105::2105*S267A and *E. coli* C41(DE3)/pET28a-NGO2105 and C41(DE3)/pET28a induced with 1 mM IPTG were harvested by centrifugation, and the pellets were washed and resuspended in PBS with 1% BSA and 0.01% Tween 20 to an OD_600_ of 0.5. These cell suspensions were incubated with anti-NGO2105 antibody (1:100) for 1 h at room temperature. Wild-type FA1090 and *E. coli* C41(DE3)/pET28a-NGO2105 incubated with preimmune serum (PI) were used as controls. After three washes in PBS, the cells were incubated with FITC-conjugated goat anti-mouse IgG antibody (1:200) in the dark for 1 h at room temperature. Untreated FA1090 and *E. coli* C41(DE3) were used as blank controls. The cells were then washed three times with PBS and analyzed using a FACS Calibur flow cytometer (Beckman Coulter).

### Analysis of the Hbp Passenger Domain Surface Exposure in *E. coli*

After 1 mM IPTG-induced expression overnight, *E. coli* C41(DE3)/pET28a-Hbp passenger-NGO2105β, *E. coli* C41(DE3)/pET28a-Hbp passenger and *E. coli* C41(DE3)/pET28a cells were collected by centrifugation, washed and resuspended in PBS with 1% BSA and 0.01% Tween 20. The anti-Myc monoclonal antibody (1:100) (Proteintech Group, Inc., Wuhan, China) was used as the primary antibody, and FITC-conjugated goat anti-mouse IgG (1:200) (Proteintech Group, Inc., Wuhan, China) was used as the secondary antibody. After three washes with PBS, cells were analyzed by flow cytometry analysis and immunofluorescence microscopy.

### Adherence and Invasion Assays

Gonococcal adherence and invasion assays were performed using human cervical carcinoma (ME-180) cells (ATCC HTB33) as described previously (Semchenko et al., 2017). Briefly, ME-180 cells were cultured in MEM medium (10% FBS) in 24-well tissue culture plates for 24 h until a confluent cell monolayer was formed. Piliated *N. gonorrhoeae* cells were inoculated onto GCB plates, grown overnight and suspended in MEM medium. The ME-180 cells were washed three times with PBS, and the prepared bacterial suspension was added to ME-180 cells at an MOI of 10:1. The inoculated dose of bacteria was confirmed by serial dilution and plating. For the adherence assays, the ME-180 cells were incubated for 1 h at 37°C in 5% CO_2_ and washed three times with PBS to remove nonadherent bacteria. The ME-180 cells were lysed with 1% saponin, and the lysates were serially diluted and then plated on GCB plates to count bacterial colony-forming units (CFUs) of the bacteria (including adherent and invasive bacteria). The adhesion rate was calculated using the ratio of cell-associated CFUs to the initial CFUs in the assay. For the invasion assays, the extracellular adherent bacteria were killed by treatment with gentamicin (100 μg/mL) for 30 min, and the cells were then washed three times with PBS and lysed with 1% saponin. The lysates were serially diluted and then plated on GCB plates to count the CFUs of the bacteria (invasive bacteria). All the experiments were performed in triplicate. The invasion rate was calculated based on the ratio of the CFUs of invasive bacteria to the initial CFUs present in the assay.

Assays of antibody-mediated adhesion inhibition were performed using ME-180 cells as described previously (Semchenko et al., 2017). Wild-type FA1090 was preincubated for 30 min with heat-inactivated anti-NGO2105 and anti-NGO2105P antiserum in MEM medium at room temperature, and heat-inactivated preimmune serum (PI) was used as the control. The preincubated bacteria were then added to the monolayer of ME-180 cells in 24-well tissue culture plates, and the subsequent steps were the same as those used in the above-described adhesion assay.

### Gonococcal Colonization in the Reproductive Tract of the Lower Genital Tract of Female Mouse

All animal use protocols were approved by the Ethics Committee at Zunyi Medical University. Eight 6- to 8-week-old female mice were randomly assigned to each group, and the mice were subcutaneously injected with 0.5 mg of the sesame oil-soluble form of estradiol on days -2, 0 (the day of bacterial challenge), and +2 to increase susceptibility to *N*. *gonorrhoeae* (Song et al., 2008; Oh et al., 2019). Antibiotics were given to prevent the overgrowth of symbiotic flora as described previously (Jerse et al., 2011). The mice in the various groups were inoculated vaginally with 2 × 10^6^ CFUs of piliated *N*. *gonorrhoeae* FA1090, FA1090Δ*ngo2105*, and FA1090Δ*ngo2105::2105* and wild-type (WT) FA1090 preincubated with a 1:20 dilution of heat-inactivated anti-NGO2105 antiserum. On days 3, 4, 5, and 6 after inoculation, the vagina was rinsed with 50 μL of normal saline for collection of the vaginal washes. The number of bacteria in these vaginal washes was counted by serial dilutions and plating on GCB plates. The results are expressed as the log10 CFUs of vaginal washes (± SD).

### Statistical Analysis

The differences between groups were analyzed using one-way ANOVA followed by Dunnett’s test. The recovery (mean log10 CFUs ± SD) of *N. gonorrhoeae* was compared by two-way ANOVA. All statistical analyses were performed using Graph-Pad Prism 5 software.

## Results

### Sequence Analysis, Expression, and Antiserum Preparation of NGO2105

NGO2105 shares amino acid sequence similarity with the Hap protein of *Hemophilus influenzae* (54% identity) and the App of *N. meningitidis* (93% identity). NGO2105 contains the typical serine autotransporter structure domain: an N-terminal signal peptide, a passenger domain (containing a peptidase S6 domain), and a C-terminal translocator domain (β-barrel). The conserved serine protease motif (GDSGSP) is present in the peptidase S6 domain of NGO2105 (Figure 1A), and forms the active site of the serine protease. H_115_, D_158_, and S_267_ comprise the predicted serine protease catalytic triad, and _953_NTL_955_ and _1189_NSG_1191_ are the predicted cleavage sites (Figure 1C). Furthermore, homology modeling showed that NGO2105 had a three-dimensional structure similar to that of the *Haemophilus influenzae* Hap protein (Meng et al., 2011; Figure 1B). Based on these *in silico* analyses, we speculated that NGO2105 might be a serine protease autotransporter in *N. gonorrhoeae*. To prepare the antiserum of the NGO2105 and passenger domain proteins, we performed preparations of expressed proteins using the *E. coli* expression system. The full-length NGO2105 protein was expressed in the form of inclusion bodies and was obtained through the washing, dissolution and affinity purification of inclusion bodies (Supplementary Figures S1A,B). The NGO2105 passenger domain was expressed in soluble form and was obtained by affinity purification (Supplementary Figure S1C). After immunization, the titers of anti-NGO2105 antiserum and anti-NGO2105 passenger antiserum in mice reached 5 × 10^6^.

### NGO2105 Is Localized and Secreted to the Outer Membrane

**FIGURE 2:**
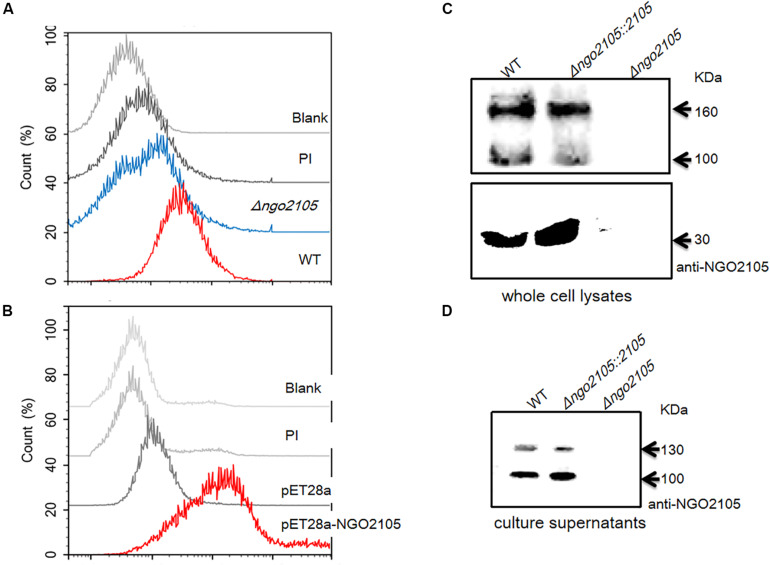
Surface localization analysis of NGO2105. **(A)** Flow cytometry analysis of the surface localization of NGO2105 in *N. gonorrhoeae*. *N. gonorrhoeae* FA1090 and FA1090Δ*ngo2105* were incubated with anti-NGO2105 antibody and stained with FITC-conjugated goat anti-mouse IgG antibody. FA1090 incubated with preimmune serum (PI) was used as a negative control, and untreated FA1090 was used as a blank control. **(B)** Flow cytometry analysis of the surface localization of NGO2105 in *E. coli*. *E. coli* C41(DE3)/pET28a-NGO2105 and *E. coli* C41(DE3)/pET28a induced with 1 mM IPTG for 4 h were incubated with anti-NGO2105 antibody and stained with FITC-conjugated goat anti-mouse IgG antibody. *E. coli* C41(DE3)/pET28a-NGO2105 incubated with preimmune serum (PI) was used as a negative control, and untreated *E. coli* C41(DE3) was used as a blank control. **(C)** Western blot analysis of whole cell lysates of wild-type (WT) FA1090, FA1090Δ*ngo2105*, and FA1090Δ*ngo2105::2105* using polyclonal anti-NGO2105 antiserum. **(D)** Western blot analysis of precipitated supernatant proteins of wild-type (WT) FA1090, FA1090Δ*ngo2105*, and FA1090Δ*ngo2105::2105* using polyclonal anti-NGO2105 antiserum.

To test whether NGO2105 is an autotransporter in *N. gonorrhoeae*, we first examined whether the NGO2105 protein is localized on the outer membrane. A flow cytometry analysis demonstrated that NGO2105 was expressed *in vivo* and is exposed on the surface of wild-type *N. gonorrhoeae* FA1090 cells, whereas no surface exposure was detected in FA1090Δ*ngo2105* mutant cells (Figure 2A). Furthermore, to verify whether the surface exposure of NGO2105 is independent of other specific factors in *N. gonorrhoeae*, the expression vector pET28a-NGO2105 was transformed into *E. coli* C41(DE3). A flow cytometry analysis showed that NGO2105 is exposed on the surface of the C41(DE3)/pET28a-NGO2105 strain, whereas no surface exposure was detected in the C41(DE3)/pET28a strain (Figure 2B). Western blot analysis showed that three bands with molecular masses of ∼160, 100, and 30 kDa were detected in the whole-cell lysates of the *N. gonorrhoeae* FA1090 and FA1090Δ*ngo2105::2105* strains using anti-NGO2105 antiserum, which suggested that these bands corresponded to the full-length NGO2105 protein, the passenger domain and the β-barrel of NGO2105, respectively. Similar results were obtained with the *N. meningitidis* App protein (Hadi et al., 2001; Serruto et al., 2003). However, none of the above-mentioned bands were detected in the FA1090Δ*ngo2105* mutant strain (Figure 2C). A Western blot analysis of precipitated proteins in the supernatant of log-phase *N. gonorrhoeae* cells was performed, and a band with a molecular mass of ∼100 kDa and a faint band with a molecular mass of ∼130 kDa were detected in the wild-type FA1090 and complemented FA1090Δ*ngo2105::2105* cells but not in the FA1090Δ*ngo2105* mutant cells (Figure 2D). These two bands correspond to the passenger domain processed by the two predicted cleavage sites: _954_NTL_956_ and _1190_NSG_1192_. Together, these results suggested that NGO2105 is exported to the outer membrane, cleaved and released in the culture supernatant, which is consistent with the characteristics of autotransporters.

### Serine 267 Is Critical for the Autoproteolytic Cleavage of NGO2105 Needed for Its Secretion

**FIGURE 3:**
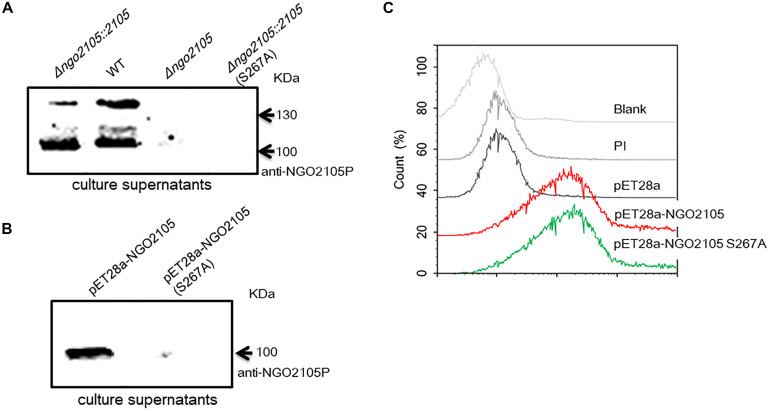
Effect of the S267A point mutation on NGO2105 secretion. **(A)** Western blot analysis of the precipitated culture supernatant samples from *N. gonorrhoeae* FA1090 (WT), FA1090Δ*ngo2105*, FA1090Δ*ngo2105::2105* and FA1090Δ*ngo2105::2105*S267A using polyclonal anti-NGO2105 passenger (anti-NGO2105P) antiserum. **(B)** Western blot analysis of the precipitated culture supernatant samples from *E. coli* C41(DE3)/pET28a-NGO2105 and *E. coli* C41(DE3)/pET28a-NGO2105 S267A using polyclonal anti-NGO2105P antiserum. **(C)** Flow cytometry analysis of the effect of the S267A point mutation on NGO2105 secretion from *E. coli*. *E. coli* C41(DE3)/pET28a-NGO2105, C41(DE3)/pET28a-NGO2105S267A and C41(DE3)/pET28a induced with 1 mM IPTG for 4 h were incubated with anti-NGO2105 antiserum and stained with FITC-conjugated goat anti-mouse IgG antibody. *E. coli* C41(DE3)/pET28a-NGO2105 incubated with preimmune serum (PI) was used as a negative control, and untreated *E. coli* C41(DE3) was used as a blank control.

To verify whether the extracellular secretion products of NGO2105 depend on its serine protease autoproteolytic activity, serine 267 of the predicted catalytic triad of the enzyme was mutated to alanine by site-directed mutagenesis (Figure 1C). A Western blot analysis showed that the same protein bands as the precipitated supernatant proteins of *N. gonorrhoeae* FA1090 were not found in the supernatant of the point mutation strain FA1090Δ*ngo2105::2105*S267A (Figure 3A). Similar results were observed with *E. coli*: no band was detected in the immunoblots of the culture supernatant proteins from *E. coli* C41(DE3)/pET28a-NGO2105S267A (Figure 3B). A flow cytometry analysis showed that NGO2105S267A was also exposed on the surface of strain C41(DE3)/pET28a-NGO2105S267A (Figure 3C), which suggested that the point mutation of S267A does not affect the surface localization of NGO2105 but only abolishes the autoproteolytic processing and secretion of NGO2105. Together, these results suggested that NGO2105 has serine protease activity and that serine 267 in the predicted catalytic triad of the enzyme is critical for the autoproteolytic processing and release of the passenger domain to the culture supernatant.

### The NGO2105 β-Barrel Might Be Involved in the Translocation of the Heterologous Passenger Domain

**FIGURE 4:**
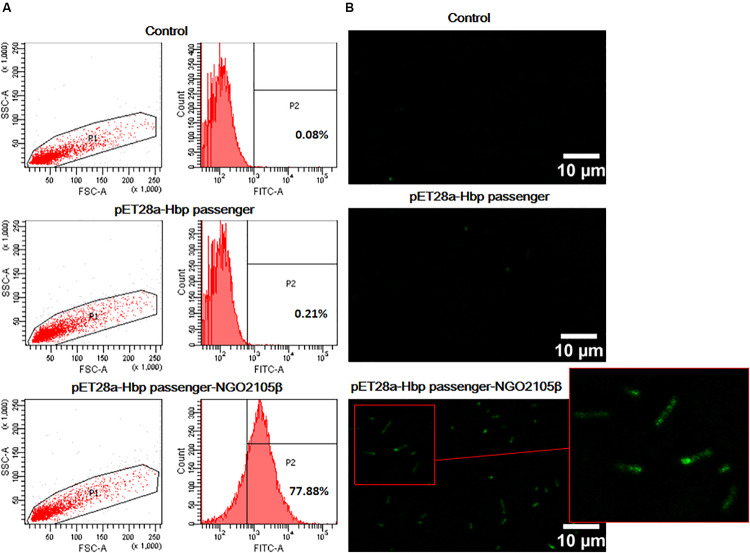
The surface display analysis of the Hbp passenger domain. *E. coli* C41(DE3) expressing Hbp passenger-NGO2105β and Hbp passenger induced with 1 mM IPTG for overnight were incubated with an anti-Myc mouse monoclonal antibody and stained with FITC-conjugated goat anti-mouse IgG antibody. *E. coli* C41(DE3)/pET28a (empty vector) was used as a negative control. Samples were analyzed by flow cytometry **(A)** and immunofluorescence microscopy **(B)**.

It is well known that some autotransporter β-barrels can transport not only their own passenger domains, but also heterologous passenger domains. To investigate whether the β-barrel of NGO2105 has a translocator function for heterologous passenger domains, we fused a modified Hbp passenger domain and the β-barrel of NGO2105 in the pET28a plasmid to construct pET28a-Hbp passenger-NGO2105β (Figure 1C). A Myc tag was added to the N-terminus of the Hbp passenger domain to facilitate determination of the surface display efficiency of the Hbp passenger domain. The pET28a-Hbp passenger-NGO2105β plasmid was transformed into *E. coli* C41(DE3) to induce expression. As a control, cells were transformed with the pET28a-Hbp passenger plasmid without the NGO2105 β-barrel. The expression and surface display of the Hbp passenger domain were assessed by flow cytometry and immunofluorescence microscopy. The flow cytometry results showed that the quantified display efficiencies on the surface of *E. coli* C41(DE3) obtained for pET28a-Hbp passenger-NGO2105β and pET28a-Hbp passenger were 77.88% and 0.21%, respectively, in comparison to the background value of 0.08% obtained with *E. coli* C41(DE3)/pET28a (Figure 4A). Immunofluorescence microscopy showed that compared to the *E. coli* C41(DE3)/pET28a-Hbp passenger showing no positive fluorescence signal, the fluorescence signal in *E. coli* C41(DE3)/pET28a-Hbp passenger-NGO2105β was obvious (Figure 4B). These results suggested that the NGO2105 β-barrel might be involved in the translocation of the heterologous passenger domain.

### NGO2105 Is Involved in Adhesion to and Invasion of Human Cervical Epithelial Cells

**FIGURE 5:**
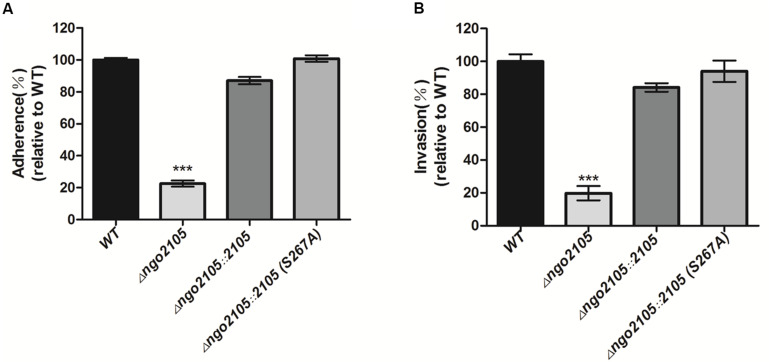
Role of NGO2105 in the adherence and invasion of *N. gonorrhoeae* to human cervical epithelial cells (ME-180). Adherence **(A)** and invasion **(B)** of *N. gonorrhoeae* FA1090 (WT), FA1090Δ*ngo2105*, FA1090Δ*ngo2105::2105*, and FA1090Δ*ngo2105::2105*S267A strains to ME-180 cells at approximately 1 × 10^6^ CFUs (MOI of 10:1). The data are shown as the average relative percentages from triplicate samples. The relative adhesion or invasion rate as calculated using the adhesion or invasion rate of each sample relative that of the wild-type strain (the adhesion or invasion rate of the wild-type strain was set to 100%). All the experiments were independently repeated three times, and one representative result (*n* = 3) is shown. Each error bar represents ± 1 SD. ***P < 0.001 for FA1090Δ*ngo2105* relative to the wild-type strain, as determined by one-way ANOVA followed by Dunnett’s test.

To investigate the biological role of NGO2105 in *N. gonorrhoeae*, we investigated the role of NGO2105 in the adhesion to and invasion of human cervical cancer cells (ME-180 cells). The FA1090Δ*ngo2105* strain exhibited 4.43-fold decreased adhesion and 5.05-fold decreased invasion compared with the wild-type strain (Figure 5). Near wild-type levels of adherence and invasion were obtained with the complemented strain. These results showed that the NGO2105 protein mediates the attachment of *N. gonorrhoeae* cells to human cervical epithelial cells *in vitro* and their subsequent invasion.

### Anti-NGO2105 and Anti-NGO2105P Antisera Are Able to Block Adherence to Cervical Epithelial Cells

**FIGURE 6:**
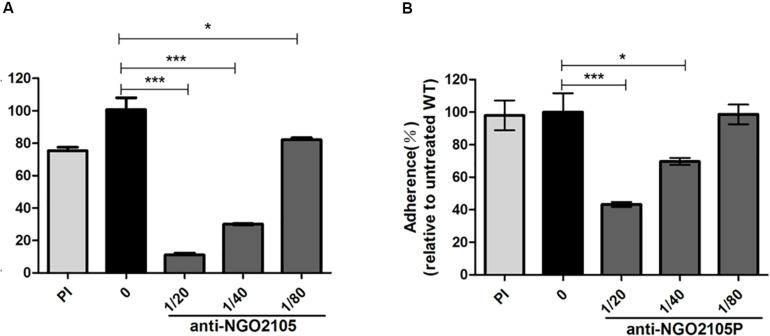
Antibody-mediated inhibition of adherence to human cervical epithelial cells (ME-180). *N. gonorrhoeae* FA1090 was preincubated with heat-inactivated anti-NGO2105, **(A)** anti-NGO2105P **(B)** antisera and preimmune (PI) serum for the adherence assay. The data are shown as the average relative percentages from triplicate samples. The relative adhesion rate was calculated using the adhesion rate of each sample relative to that of the untreated wild-type strain (0) (the adhesion rate of the untreated wild-type strain was set to 100%). All the experiments were independently repeated three times, and one representative result (*n* = 3) is shown. Each error bar represents ± 1 SD. *P < 0.05, and ***P < 0.001 relative to the untreated wild-type strain, as determined by one-way ANOVA followed by Dunnett’s test.

To further verify the role of NGO2105 in the adhesion of *N. gonorrhoeae*, we performed an antibody adhesion inhibition experiment using two antisera. Anti-NGO2105 reacted with the full-length NGO2105 protein, and anti-NGO2105P reacted with the passenger domain of NGO2105. The ability of *N. gonorrhoeae* to adhere to ME-180 cells could be inhibited in a concentration-dependent manner by preincubation of the *N. gonorrhoeae* FA1090 strain with anti-NGO2105 antiserum. When the anti-NGO2105 antiserum was diluted 1:20, 1:40, and 1:80, the inhibition level of the *N. gonorrhoeae* FA1090 strain was 9. 15-, 3. 35-, and 1.67-fold lower than that of the untreated wild-type strain, respectively (Figure 6A). Similarly, when the anti-NGO2105P antiserum was diluted 1:20 and 1:40, the inhibition level of *N. gonorrhoeae* was 2.31- and 1.43-fold lower than that of the untreated wild-type strain, respectively, and no significant difference was found between the serum at 1:80 dilution and the preimmunized serum (Figure 6B). These results showed that both anti-NGO2105 and anti-NGO2105P antisera can significantly inhibit the adhesion of *N. gonorrhoeae*.

### The *ngo2105* Mutant and Anti-NGO2105 Antiserum Significantly Attenuate the Colonization of *N. gonorrhoeae* in Mice

**FIGURE 7:**
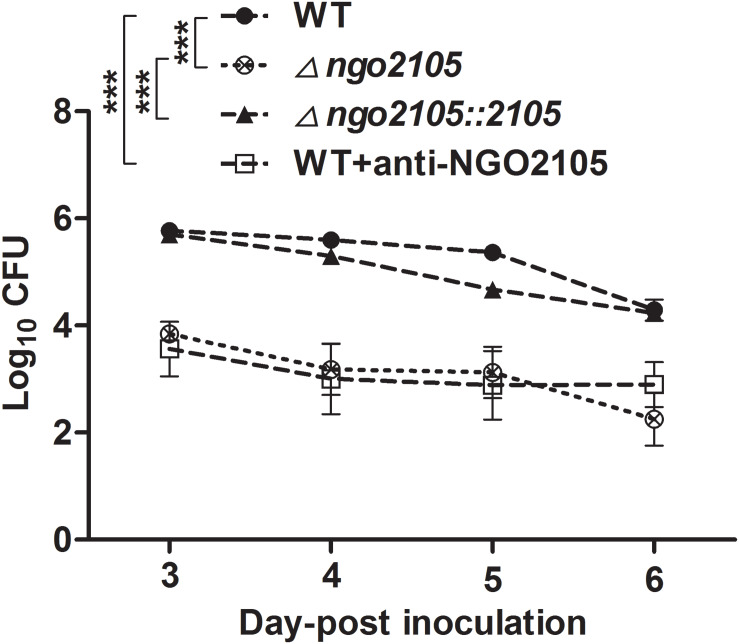
Role of NGO2105 in *N. gonorrhoeae* colonization in the mouse lower genital tract. The data are expressed as the mean log10 CFUs of vaginal washes (± SD, *n* = 8) at 3, 4, 5, and 6 days after infection with wild-type (WT) FA1090, FA1090Δ*ngo2105*, FA1090Δ*ngo2105::2105*, and FA1090 cells preincubated with heat-inactivated anti-NGO2105 antiserum at 1:20 dilution as shown. The curves were analyzed by two-way ANOVA. ***P < 0.001.

For investigation of the effect of the *ngo2105* deletion mutant on infection, we used a female mouse model of lower genital tract infection to test the effect of the *ngo2105* mutant on colonization *in vivo*. The mice were inoculated intravaginally with a suspension containing similar numbers of wild-type FA1090, FA1090Δ*ngo2105*, and FA1090Δ*ngo2105::2105* cells and wild-type FA1090 cells preincubated with a 1:20 dilution of heat-inactivated anti-NGO2105 antiserum. Vaginal secretions were then collected, and the number of colonies was counted. As shown in Figure 7, the number of FA1090Δ*ngo2105* cells in vaginal washes was significantly lower than that of wild-type FA1090. The complement strain FA1090Δ*ngo2105::2105* could restore the colonization ability to near wild-type levels. Pretreatment with anti-NGO2105 antiserum significantly inhibited the colonization ability of wild-type FA1090 cells. These results suggested that the NGO2105 protein plays an important role in the colonization of *N. gonorrhoeae*.

## Discussion

In this study, we identified NGO2105, a new autotransporter in *N. gonorrhoeae* and our analyses revealed the following: (1) NGO2105 is exported to the outer membrane, cleaved and released in the culture supernatant, (2) NGO2105 has serine protease activity, and (3) serine 267 in the predicted catalytic triad of the enzyme is critical for the autoproteolytic cleavage needed for secretion. The NGO2105 β-barrel also has the ability to translocate the heterologous Hbp passenger domain. *N. gonorrhoeae* lacking *ngo2105* exhibited markedly reduced adherence to and invasion into human cervical epithelial cells, and both anti-NGO2105 and anti-NGO2105P antisera significantly inhibited the adhesion of *N. gonorrhoeae*. The Δ*ngo2105* mutant and anti-NGO2105 antiserum significantly attenuated the colonization of *N. gonorrhoeae* in mice.

Several autotransporter proteins have previously been identified in *N. meningitidis*, and these include IgA1 protease, Nalp, App, MspA, AutA, AutB, NadA, and NahhA (Ait-Tahar et al., 2000; Hadi et al., 2001; Turner et al., 2002, 2006). The IgA1 protease of *N. gonorrhoeae* was the first autotransporter identified, and the IgA1 protease is also the only autotransporter identified in *N. gonorrhoeae* (Pohlner et al., 1987). Using common molecular features of autotransporter proteins, our *in silico* analyses predicted that NGO2105 possesses the typical domain characteristics of autotransporter proteins. We initially identified a three-dimensional structure of NGO2105 that was similar to that of the Hap autotransporter protein. Moreover, the NGO2105 protein exhibited a high degree of sequence homology with App, an adhesion and penetration protein in *N. meningitidis* that has been classified as a chymotrysin-like serine protease (Hadi et al., 2001). According to a flow cytometry analysis, we found that the NGO2105 protein is expressed and exported to the bacterial surface, and these processes are similar to those that occur in *E. coli*, which indicates that these processes are independent of the other specific gonorrhoeae factors. The probing of whole-cell lysates of *N. gonorrhoeae* FA1090 with the NGO2105 antiserum detected strong bands at 160 kDa (full-length protein) and 30 kDa (β-barrel) and weaker bands at 100 kDa (partial passenger domain). This result differs from that obtained for App from *N. meningitidis*. The 175, 160, 140, and 100 kDa bands were detected with the *N. meningitidis* MC58 strain, but the β-barrel band was not detected in the whole bacterial lysates (Hadi et al., 2001). The difference might be related to the different proteolysis of the two proteins between the two microorganisms. The passenger domains of some serine protease autotransporters are released into the extracellular environment by autoproteolytic cleavage or by the cleavage of another autotransporter. In *N. meningitidis*, two autotransporters, App and IgA1 protease, are processed by autoproteolytic processing and can also be cleaved by the autotransporter NalP (van Ulsen et al., 2003; Roussel-Jazede et al., 2014). Notably, *N. gonorrhoeae* does not produce NalP protein because the gene is disrupted (van Ulsen and Tommassen, 2006), which might explain the difference between the immunoblotting bands of NGO2105 and those of App. Based on analogy to the known cleavage sites of autotransporters such as Hap and App (Fink et al., 2001; Serruto et al., 2003), two cleavage sites could be predicted within the NGO2105 amino acid sequence: _954_NTL_956_ and _1190_NSG_1192_. After processing at residue 1190, the obtained fragment has a predicted molecular weight of 125.31 kDa, whereas after cleavage at position 954, the obtained fragment has a predicted molecular weight of 99.38 kDa. These two predicted fragments might match up with the two bands of approximately 130 and 100 kDa observed in the *N. gonorrhoeae* culture supernatant. However, only the 100 kDa fragment was detected in the culture supernatant from the *E. coli* C41(DE3)/pET28a-NGO2105 strain, which might suggest that the proteolytic cleavage of NGO2105 prefers to occur at _954_NTL_956_ in *E. coli* C41(DE3). A similar result was also observed when App was expressed in *E. coli* (Serruto et al., 2003). The serine protease activity of NGO2105 was confirmed by mutating the serine at position 267 to alanine, which abolished the processing and secretion of the passenger domain in the *N. gonorrhoeae* and *E. coli* strains. In NGO2105, serine 267 belongs to a catalytic triad together with histidine 115 and aspartate 158, and all three catalytic residues are conserved in the App protein and other autotransporter proteins (Fink et al., 2001). Based on these results, NGO2105 could be classified as a serine protease autotransporter protein in *N. gonorrhoeae*.

Various specific β-barrels of autotransporters have been shown to secrete recombinant proteins to the cell surface, but the transport efficiencies of different β-barrels are different. The Hbp passenger domain is often used as a transport target to evaluate the transport capacity of the β-barrels of autotransporters (Jong et al., 2018). To investigate whether the NGO2105 β-barrel can secrete the heterologous passenger domain, we fused a small Myc tag and Hbp passenger domain upstream of the β-barrel of NGO2105. To address whether the Hbp passenger-NGO2105β constructs were expressed and targeted to the outer membrane, the surface localization of the fusion protein Hbp passenger-NGO2105β was analyzed by flow cytometry and immunofluorescence microscopy. Significant surface localization could be detected on the surface of *E. coli* C41/pET28a-Hbp passenger-NGO2105β cells, but no significant surface localization was found on the surface of *E. coli* C41/pET28a-Hbp passenger cells. These results suggested that the β-barrel of NGO2105 might be involved in the translocation of the heterologous passenger domain.

NGO2105 exhibits a high degree of homology with Hap and App; Hap has been implicated in *H. influenzae* colonization of the respiratory mucosa, and App mediates the adhesion of *N. meningitidis* to Chang cells (Fink et al., 2003; Serruto et al., 2003). *N. gonorrhoeae* can cause infection in different mucous tissues, such as the urethra, cervix, fallopian tube, rectum, nasopharynx, and conjunctiva. Adhesion and internalization are important links in the establishment of local mucosal infection. Type IV pili mediate initial adhesion to the surface of mucosal cells, and after this step, opaque-associated proteins and additional adhesins and invasins drive adhesion and internalization (Virji et al., 1991; Rudel et al., 1995; Virji et al., 1996). We evaluated whether NGO2105 is involved in cell adhesion and invasion. The *ngo2105-*knockout strain exhibited impairments in its abilities to adhere to and invade ME-180 epithelial cells compared with those of the wild-type strain. As expected, we found that anti-NGO2105 and anti-NGO2105P antisera are able to reduce *N. gonorrhoeae* adherence to ME-180 epithelial cells. These results showed that NGO2105 played an important role in the adhesion and invasion of *N. gonorrhoeae* to ME-180 epithelial cells. We also found that the inhibition efficiency of anti-NGO2105P was significantly lower than that of anti-NGO2105, which suggested that both the NGO2105β domain and the secreted passenger domain might be involved in the processes of adhesion and invasion. A similar result was also observed for the *N. meningitidis* App protein. *E. coli* strains expressing recombinant App β-barrel and App passenger domain constructs are able to bind to Chang cells, and their adhesion ability is significantly lower than that of full-length App (Serruto et al., 2003).

Previous studies have shown that serine protease autotransporters can target a wide range of leukocyte glycoproteins and cleave mucin family substrates, which might provide additional advantages for pathogens (Ruiz-Perez et al., 2011). Pic protease, a serine protease transporter found in *Shigella*, can induce mucus release and cleave mucin, which is beneficial to its colonization in mucosa (Harrington et al., 2009). Here, we evaluated the role of NGO2105 in *N. gonorrhoeae* colonization using a gonorrhea genital tract infection model. We provide *in vivo* evidence showing that NGO2105 plays a role in the process of *N. gonorrhoeae* colonization and that an anti-NGO2105 antibody can inhibit the colonization of *N. gonorrhoeae*, which suggests that it might be a potential protein vaccine target.

## Conclusion

In summary, we report the functional characterization of the *N. gonorrhoeae* NGO2105 protein as a serine protease autotransporter and evaluated its role in host–pathogen interactions. The present study increases our knowledge concerning the role of NGO2105 in the physiology and pathogenesis of *N. gonorrhoeae*. As our next step, we need to further screen the receptor of NGO2105 in human cervical epithelial cells to better understand the mechanism of NGO2105 in the pathogenesis of *N. gonorrhoeae*. In addition, whether NGO2105, similarly to APP, can be internalized and trafficked to the nucleus of human cervical epithelial cells also needs to be determined. Further experimental evidence is needed to assess the potential of NGO2105 as a protein vaccine target.

## Data Availability Statement

All datasets generated for this study are included in the article/Supplementary Material.

## Ethics Statement

The animal study was reviewed and approved by the Ethics Committee at Zunyi Medical University.

## Author Contributions

JH, QZ, MH, and XM designed the experiments, analyzed the data, and wrote the manuscript. JH, QZ, JC, ZuC, JY, and YW performed the experiments and analyzed the data. TZ, ZeC, and ZM contributed to the reagents and materials and analyzed the data. All authors read and approved the final manuscript.

## Conflict of Interest

The authors declare that the research was conducted in the absence of any commercial or financial relationships that could be construed as a potential conflict of interest.

## Footnotes

1 http://swissmodel.expasy.org
