# Supplementary material for: Neisseria gonorrhoeae NGO2105 Is an Autotransporter Protein Involved in Adhesion to Human Cervical Epithelial Cells and in vivo Colonization
Source: Front Microbiol. 2020 Jun 25;11:1395. doi: 10.3389/fmicb.2020.01395 (PMC7330057; doi:10.3389/fmicb.2020.01395)
Supplement: Supplementary file 1 [file Data_Sheet_1.PDF]

Supplementary Material

1.1 Supplementary Figures

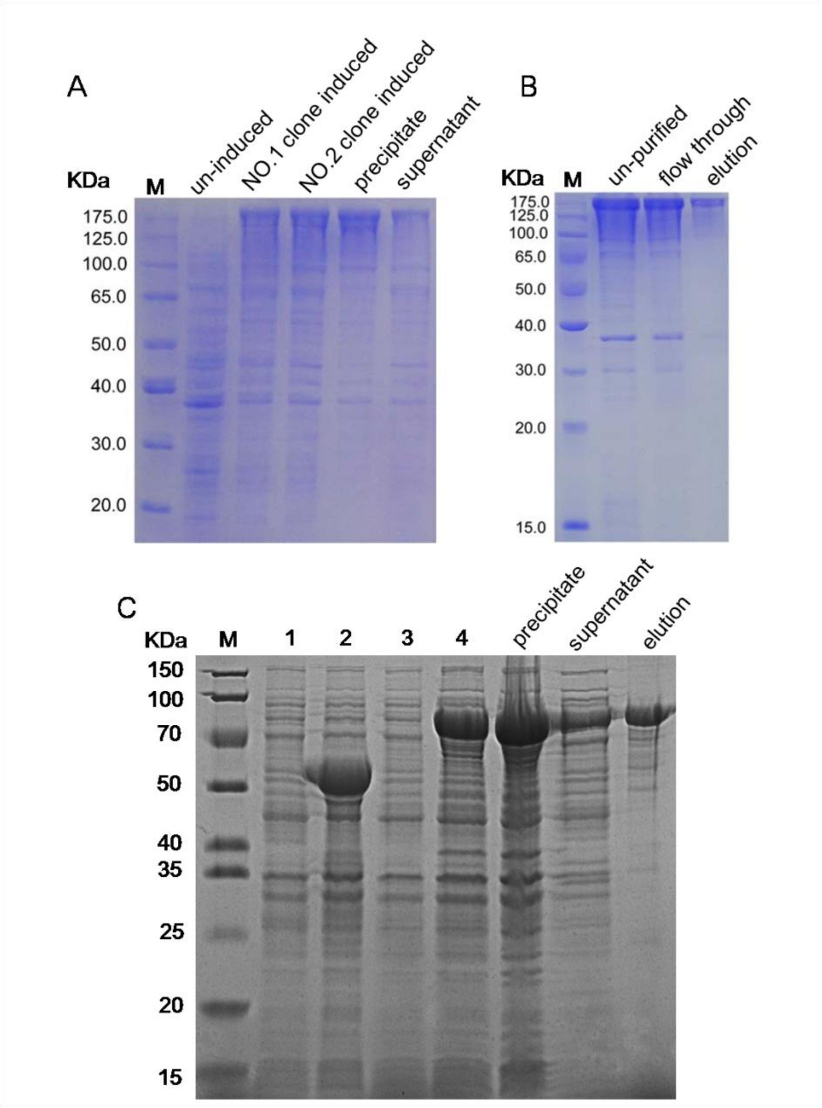

Supplementary Figure 1. Expression and purification of recombinant NGO2105 and NGO2105 passenger domain (NGO2105P). A. SDS-PAGE of recombinant NGO2105 expression in *E. coli*. B. SDS-PAGE of purification recombinant protein NGO2105. C. SDS-PAGE of recombinant NGO2105P expression in *E. coli* and protein purification. The un-induced *E.coli* with the pCold TF and pCold TF-NGO2105P vectors (Lane 1 and 3). 0.2mM IPTG induced *E.coli* with the pCold TF and pCold TF-NGO2105P vectors (Lane 2 and 4).
